# Musical Museum: An Interdisciplinary Approach to Integrative Life Enrichment for Individuals with Neurocognitive Disorders and their Care Partners

**DOI:** 10.1002/alz70858_100035

**Published:** 2025-12-25

**Authors:** Melanie Zhang, Jonathan Zhao, Kate Vidano, Clara Takarabe, Borna Bonakdarpour

**Affiliations:** ^1^ Mesulam Center for Cognitive Neurology and Alzheimer's Disease, Chicago, IL, USA; ^2^ Northwestern University Feinberg School of Medicine, Chicago, IL, USA; ^3^ Northwestern Music and Medicine Program, Chicago, IL, USA; ^4^ Northwestern University, Evanston, IL, USA

## Abstract

**Background:**

Social, emotional, and cognitive stimulation are protective factors that can slow neurocognitive disorder (NcD) progression, making them priorities for early and moderate dementia interventions. Live music programs with music appreciation content provide social‐emotional interaction, intellectual stimulation, and serve as social prescriptions. However, their application in NcD remains underexplored. This abstract introduces *Musical Museum*, a concert curriculum blending live performances with intellectually stimulating content in a supportive environment. Survey results from individuals with NcD and their care partners are also presented, highlighting the program's potential benefits.

**Method:**

*Musical Museum* participants included individuals with NcD and their care partners. Each 45‐60 minute session featured a combination of familiar music based on participants’ reminiscence bumps and preference surveys, as well as less familiar, simple, diatonic, culturally diverse music. Participants were provided printed programs featuring simple language, including poetry and lyrics where applicable. Verbal introductions, highlighting musical facts and historical context, were crafted for intellectual engagement without overwhelming participants. A post‐program reception facilitated interaction, and surveys assessed satisfaction, intellectual stimulation, and mood changes, analyzed using a Wilcoxon signed‐rank test.

**Result:**

The number of participants increased from 27 to 64 as the series progressed, with a total of 220 participants completing surveys across 10 sessions. On a five‐point Likert scale, respondents reported an overall satisfaction of 4.4, an average pleasure rating of 4.3, intellectual stimulation level of 4.1, and likelihood to recommend of 4.7. Respondents also indicated a significant improvement in mood post‐session (4.5) compared to pre‐session (3.4), as measured by a Wilcoxon test (*p* = 0.014; *W* = 0).

**Conclusion:**

The *Musical Museum* series demonstrated feasibility and strong reception, evidenced by the growing attendance and positive survey feedback. Participants’ positive feedback highlighted the potential of live music programs to enhance social‐emotional interaction and cognitive engagement, consistent with the goals of social prescription. These findings support the value of engagement with music as a multimodal intervention in NcD care. Future efforts will focus on incorporating real‐time neurophysiologic measures and expanding the collection of audience response data, further characterizing the impact of such interventions.

